# Heparin binding protein in severe COVID-19—A prospective observational cohort study

**DOI:** 10.1371/journal.pone.0249570

**Published:** 2021-04-06

**Authors:** Lisa Mellhammar, Louise Thelaus, Sixten Elén, Jane Fisher, Adam Linder

**Affiliations:** Faculty of Medicine, Department of Clinical Sciences Lund, Division of Infection Medicine, Lund University, Lund, Sweden; Heidelberg University Hospital, GERMANY

## Abstract

**Background and aims:**

Neutrophil-derived heparin binding protein (HBP; also known as azurocidin or CAP-37) is a key player in bacterial sepsis and a promising biomarker in severe infections.

The aims of this study were to assess whether HBP is involved in the pathophysiology of COVID-19 and, if so, whether it can be used to predict severe disease preferably using a point-of-care test.

**Methods:**

This was a prospective convenience sample study of biomarkers in patients admitted to Skåne University hospital in Sweden with a confirmed COVID-19 diagnosis. Plasma samples and clinical data were collected within 72h after admission, during hospital stay and at discharge. Plasma HBP concentrations samples were measured both with enzyme-linked immunosorbent assay (ELISA) and with a novel dry immunofluorescence analyzer (Joinstar) point-of-care test.

**Results:**

Thirty-five COVID-19 patients were enrolled in the study. Twenty-nine patients had blood samples taken within 72h after admission. We compared the highest HBP value taken within 72h after admission in patients who eventually developed organ dysfunction (n = 23) compared to those who did not (n = 6), and found that HBP was significantly elevated in those who developed organ dysfunction (25.0 ng/mL (interquartile range (IQR) 16.6–48.5) vs 10.6 ng/mL (IQR 4.8–21.7 ng/mL), *p* = 0.03). Point-of-care test measurements correlated well with ELISA measurements (R = 0.83). HBP measured by the POC device predicted development of COVID-induced organ dysfunction with an AUC of 0.88 (95% confidence interval (CI) 0.70–1.0).

**Conclusions:**

HBP is elevated prior to onset of organ dysfunction in patients with severe COVID-19 using a newly developed point-of-care test and hence HBP could be used in a clinical setting as a prognostic marker in COVID-19.

## Introduction

By December 8^th^ 2020, the number of confirmed cases of coronavirus disease (COVID-19) was more than 65.8 million worldwide [1]. Most patients experience a mild illness, but 5% develop sepsis [2]. Sepsis is a life-threatening organ dysfunction caused by a dysregulated host response to infection. Although sepsis is most often caused by bacteria, other pathogens like fungi or viruses can cause it to a lesser extent [3]. Patients with severe COVID-19 often have a dysregulated host response with evident hyperinflammation and immunosuppression, even though the majority of COVID-19 patients with sepsis do not have a bacterial superinfection [4–6]. Sepsis is a heterogenous disease and the pathophysiology of sepsis due to COVID-19 is not fully understood [7, 8].

Neutrophils are a key player in bacterial sepsis, but their role in viral sepsis is less well defined [9]. Patients with severe COVID-19 tend to have elevated numbers of neutrophils in the blood and in the lungs, suggesting that they might be involved in COVID-19 pathophysiology [10, 11]. Heparin binding protein (HBP; also known as azurocidin or CAP-37), is a neutrophil protein that plays an important role in bacterial sepsis [12]. HBP is stored in the secretory vesicles and azurophilic granules of neutrophils and is released upon activation [12]. Because it is prefabricated and released early during infections, HBP is a promising biomarker in severe infections. Plasma HBP levels are elevated up to 12 hours before the first signs of circulatory failure and organ dysfunction appear in patients with sepsis [13]. HBP is also a strong inducer of endothelial barrier dysfunction and inflammation and predicts lung and kidney dysfunction in patients with sepsis and septic shock [14–16]. Although best studied in bacterial infections, elevated HBP levels have been found in some severe viral infections including Influenza A (H1N1) and COVID-19 [17, 18].

Several studies have suggested that SARS-COV-2 infection might disproportionately affect endothelial cells and that pre-existing endothelial damage due to aging, cardiovascular diseases or diabetes mellitus could lead to more severe COVID-19 [19–21]. The endothelial derangements in severe COVID-19 and HBP’s role in endothelial dysfunction makes HBP interesting to study both as a potential prognostic marker and as a target molecule for therapy in severe COVID-19.

The primary aims of this study were to assess whether HBP can predict sepsis in COVID-19 and if point-of-care (POC) testing can substitute more time-consuming analysis with enzyme-linked immunosorbent assay (ELISA).

## Materials and methods

### Sample and clinical data collection

Patients ≥18 years admitted to the clinic for infectious diseases at Skåne University Hospital (Lund, Sweden) with a PCR-confirmed COVID-19 diagnosis were enrolled prospectively in a convenience study of biomarkers, referred to as the “COVID-19 cohort”. Patients were included from the 20^th^ of May 2020 to the 28^th^ of June 2020. The only exclusion criteria were difficulties in understanding the meaning of participation, and lack of consent. EDTA-blood was collected from patients and centrifuged for 10 minutes at 2000 xg within 60 minutes from collection. Plasma was separated and stored at -80 °C until further analysis. Samples were collected within 72 hours after admission, at several time points during hospital stay, and at discharge for most patients due to logistical issues. Because sampling timepoints were not standardized, the number of patient samples at each time point varied. We had several samples collected in the first 72 hours, and so we used the highest HBP value measured during this time for prediction of organ dysfunction.

For comparison to bacterial sepsis and other viral sepsis, two cohorts of patients originally enrolled for prospective multicenter studies of biomarkers were used. In the first cohort, patients were included consecutively if they were ≥18 years of age and had suspected sepsis i.e., with fever or history of fever and highest priority according to Rapid Emergency Triage System (RETTS) or lactate > 3.5mmol at Skåne University Hospital in Lund between April 2017 and February 2018 (unpublished). Plasma samples was collected at ED admission as part of clinical routine. Patients who had a positive blood culture for bacteria and fulfilled the sepsis-3 criteria [3] were selected from this cohort for inclusion in the present study, and are referred to as the “bacterial sepsis cohort”. In the cohort referred to as the “viral sepsis cohort”, plasma samples from all patients with PCR-verified viral infections other than COVID-19 were selected from a cohort of ED patients. In this cohort, inclusion criteria were age ≥18 years with at least one of: respiratory rate >25 breaths/minute, heart rate >120 beats/minute, altered mental awareness, systolic blood pressure below 100 mmHg, SpO2 <90%, or <93% if ongoing oxygen treatment in February 2015 and then again from January to March 2016. The only exclusion criteria were difficulties in understanding the meaning of participation [22].

Ethical approval for each cohort was carried out by the Lund University Ethical Review Board. Written informed consent was obtained from participants in the COVID-19 cohort and the viral sepsis cohort (ethical permission no. 2020/02218 and 2014/741), while participant opt-out were applied for the bacterial sepsis cohort as approved by the Lund University Ethical Review Board (ethical permission no. 2016/271).

Data on comorbidities, organ dysfunction, treatment, intensive care and mortality were collected from medical records. Organ dysfunction was defined as a change in sequential organ failure assessment (SOFA) score of ≥2 at any time during admission.

### Analysis of biomarkers

To compare HBP levels in COVID, bacteremia and viral non-COVID-19 infection, an ELISA method was used for quantification of HBP. HBP concentrations for the bacterial and viral cohort were measured prior to this study, in the years 2015–2017, and the values were extracted from the stored data.

For analyses involving prediction of organ failure HBP concentrations were also measured with a novel Dry Immunofluorescence Analyzer (Jet-iStar 800) (Joinstar) technique to evaluate the usefulness of the new point of care device for prediction of clinical outcomes.

### ELISA

ELISA kits for measuring HBP concentration were purchased from Axis-Shield Diagnostics (catalogue no FMHBP100IUO) and used according to the manufacturer’s directions. Recommended dilutions of 1:40 were used for all plasma samples unless samples was found outside the range of the standard curve, in which case the samples were diluted further.

### Point-of-care (POC) assay

Additionally, we verified the Jet-iStar 800 point of care device (catalogue no FGCOV100), which is based on immunoassay technology, for rapid detection of HBP. This rapid test gives a result within 20 minutes and could be a more convenient method for clinical measurement of HBP. A description of the device and method is provided in the S1 File.

With the aim to assess some key analytical performance aspects of the HBP point of care assay, a verification in multiple steps was performed. Accuracy, lower limit of detection, linearity, and precision were verified to be within the ranges specified by the manufacturer using methods described in the S1 File.

### Grouping of samples

Because of logistical issues we did not collect samples at standardize time points in the COVID-19 cohort, and each patient had several samples taken during the course of hospital stay. Therefore we grouped samples that were taken within a specified timeframe. When comparing COVID-19 to bacterial and viral sepsis, “admission” values were the highest value measured within 72 hours after admission because this was the earliest available sample for most patients, but some patients had multiple samples taken during this time.

When analyzing the kinetics of HBP over time, “admission” values were the earliest HBP value recorded within the first 72h after admission. “Before organ dysfunction” values were the latest measurement taken up to 48 hours before organ dysfunction was recorded. Samples from patients who developed organ dysfunction within 72h after admission were included in the “before organ dysfunction” group. “Discharge” values were the latest measurement taken up to 48 hours before the patient was discharged.

### Statistical analysis

Independent, continuous variables are presented as median with interquartile ranges (IQR) and were analysed with using Kruskal-Wallis test with Dunn’s post-hoc test for multiple comparisons when three or more groups were compared, or Mann-Whitney test when two groups were compared. Receiver operating characteristic (ROC) curves and the area under the curve (AUC) were generated to determine the predictive value of HBP (highest measured value 72 hours from admission) for development of organ dysfunction at any time during hospital stay. Statistical analyses were performed using GraphPad Prism version 8.3.1. A *p*-value < 0.05 was considered statistically significant.

## Results

### Patient characteristics

Thirty-five patients were eligible for inclusion in the COVID-19 cohort. From 6 patients, blood samples were not taken at arrival or within 72h from admission and they were excluded from analyses of admission samples. Patient characteristics and demographics for each cohort are presented in Table 1.

**Table 1:** Characteristics of included patients.

	COVID-19 n = 29	Bacterial sepsis n = 24	Viral sepsis n = 28
**Baseline characteristics**			
Sex (female), n (%)	10 (34)	11 (46)	15 (54)
Age, median (IQR)	66 (60–77)	74 (71–79)	71 (64–84)
**Comorbidities**			
Diabetes mellitus, n (%)	4 (14)	5 (21)	4 (14)
Cardiovascular disease, n (%)	12 (41)	12 (50)	9 (32)
Renal disease, n (%)	4 (14)	3 (13)	3 (11)
Respiratory disease, n (%)	5 (17)	4 (17)	6 (21)
Malignancy, n (%)	1 (3)	4 (17)	5 (18)
**Clinical characteristics**			
SOFA increase<72h	2	2	2
Critical care, n (%)	3 (10)	5 (21)	3 (11)
Mechanical ventilation, n (%)	2 (7)	2 (8)	0 (0)
Vasopressor, n (%)	2 (7)	5 (21)	2 (7)
Acute dialysis, n (%)	1 (3)	1 (0)	0 (0)
Mortality (in-hospital), n (%)	3 (10)	2 (8)	0 (0)

SOFA, Sequential Organ Failure Assessment; IQR, interquartile range; HBP, heparin binding protein

All but two patients of the COVID-19 cohort had blood bacterial cultures taken and none were positive indicating no bacteremia in this cohort. Two patients had positive bacterial cultures in samples from the respiratory tract. One had *Staphylococcus aureus* in sputum, but it was not considered a clinical bacterial infection and the patient did not receive antibiotic therapy. One patient had polymicrobial flora in a tracheal specimen 10 days after admission and this was considered as secondary to a deterioration due to COVID-19.

### Admission HBP levels in COVID-19 and bacterial and viral sepsis

The bacterial sepsis cohort included 24 patients and the viral sepsis cohort included 28 patients.

**Fig 1:**
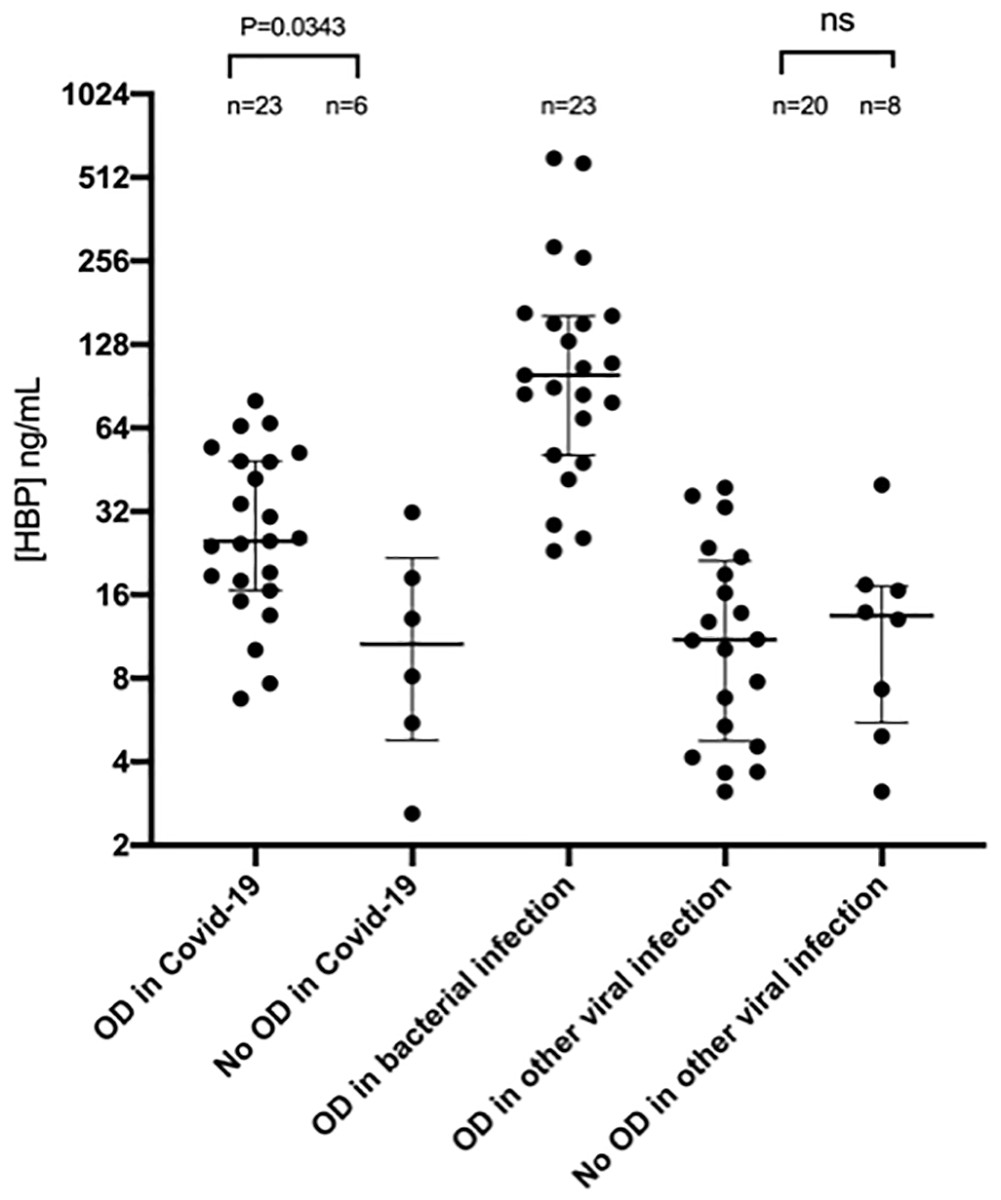
Plasma concentration of HBP in patients with severe COVID-19 and other sepsis. Plasma HBP levels measured by ELISA in patients with COVID-19, verified bacterial infection or other viral infection than COVID-19 with and without organ dysfunction (OD). Only one patient with bacterial infection did not have organ dysfunction and is not shown on the graph. HBP values in the COVID-19 cohort are the highest value measured in the first 72 hours from admission. Samples in the bacterial and other viral cohorts were taken at admission. Values were compared using Kruskal-Wallis test with Dunn’s post-hoc test for multiple comparisons.

First, HBP levels on admission (highest value measured within 72h after admission) in COVID-19 patients were compared to HBP levels on admission in patients in the bacterial sepsis and viral sepsis cohorts to determine if HBP was elevated in COVID-19 patients. Patients within each cohort were divided into two groups based on whether they developed organ dysfunction at any time. HBP on admission (highest value measured within 72h from admission) was elevated in COVID-19 patients with onset of organ dysfunction at any time (n = 23) compared to those without (n = 6), 25.0 ng/mL (IQR 16.6–48.5) vs 10.6 ng/mL (IQR 4.8–21.7 ng/mL), *p* = 0.03, while there was no difference between those who developed organ dysfunction (n = 20) and those who did not (n = 8) in the viral sepsis cohort, 11.0 ng/mL (4.8–21.2) vs 13.5 (5.5–17.2) ng/mL (*p* = 0.10) (Fig 1). In the bacterial sepsis cohort, all but one patient developed organ dysfunction, so this comparison was not possible in this cohort. Table 2.

**Table 2:** Median and interquartile range of plasma HBP concentrations in the various analysis groups. Number of patients for each group are indicated at the top, and the number of samples available for each analysis are indicated in each cell.

	COVID-19			Bacterial sepsis			Other viral sepsis		
Timepoint	All patients (n = 35)	OD (n = 30)	No OD (n = 5)	All patients (n = 24)	OD (n = 23)	No OD (n = 1)	All patients (n = 28)	OD (n = 20)	No OD (n = 8)
Admission	24.0 (13.3–45.1)^1^ (n = 29)	25.0 (16.6–48.5)^1^ (n = 23)	10.6 (4.8–21.7)^1^ (n = 6)	102.3 (55.6–165.3) (n = 22)	99.3 (51.1–162.4) (n = 23)	NA (n = 1)	11.9 (5.1–18.5) (n = 28)	11.0 (4.8–21.2) (n = 20)	13.5 (5.5–17.2) (n = 8)
Admission^2^ (n = 20)		7.6 (5.9–15.0)							
Before OD^3^ (n = 14)		22.4 (10.8–43.9)							
Discharge^4^ (n = 12)		9.0 (5.9–14.7)							

^1^Highest value measured within 72h of admission. Admission samples were unavailable from 6 patients

^2^Earliest value measured within 72h of admission, excluding patients who developed OD in the first 72h

^3^Latest value measured within 48h before onset of OD

^4^Latest value measured within 48h before hospital discharge

OD = organ dysfunction

### HBP point of care test for prediction of organ dysfunction

**Fig 2:**
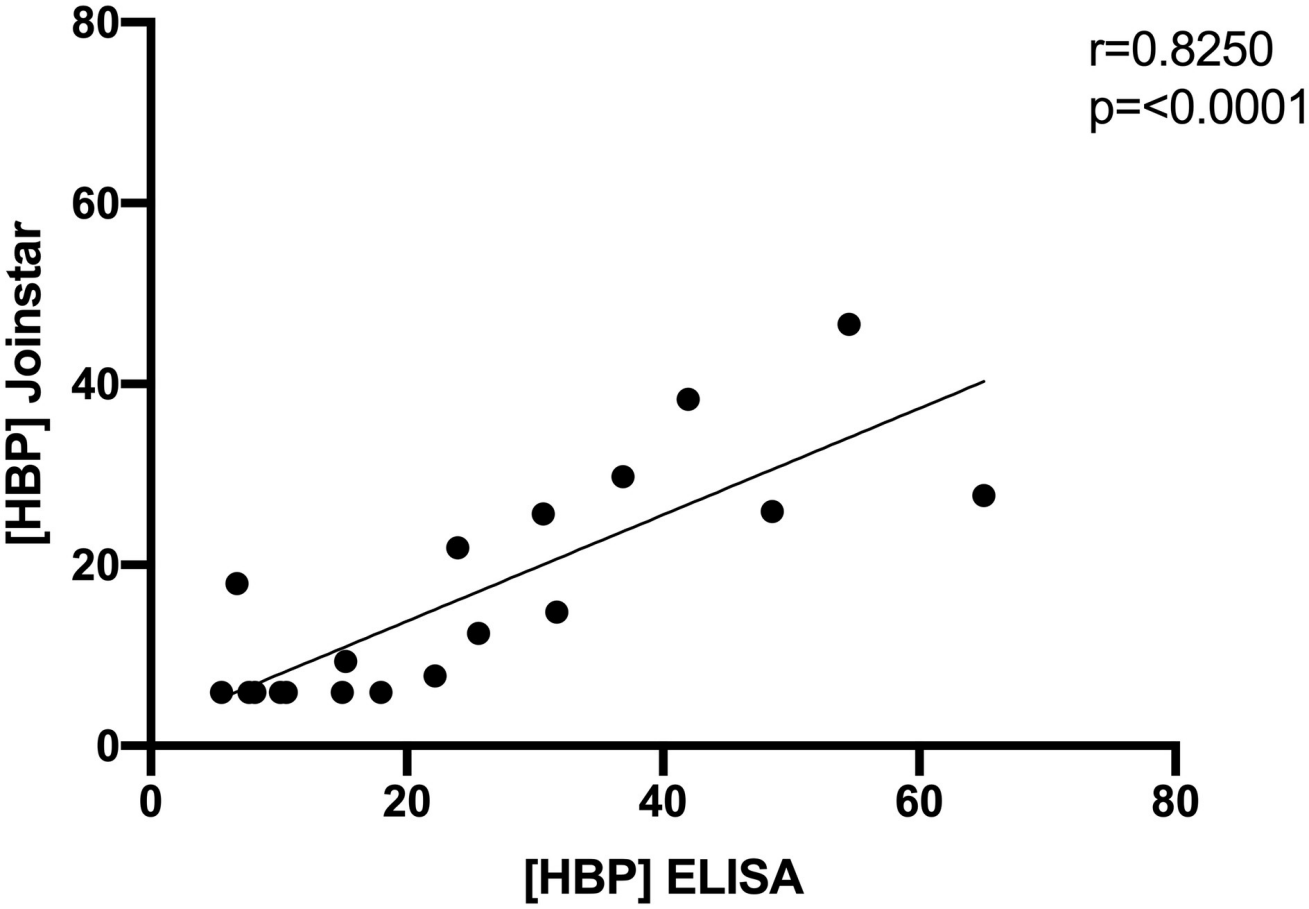
Correlation between ELISA and Joinstar values. Correlation of HBP levels in the same samples measured by Joinstar point of care device and ELISA. Correlation coefficient R = 0.8250; p<0.01). Values were compared using Spearman correlation.

**Fig 3:**
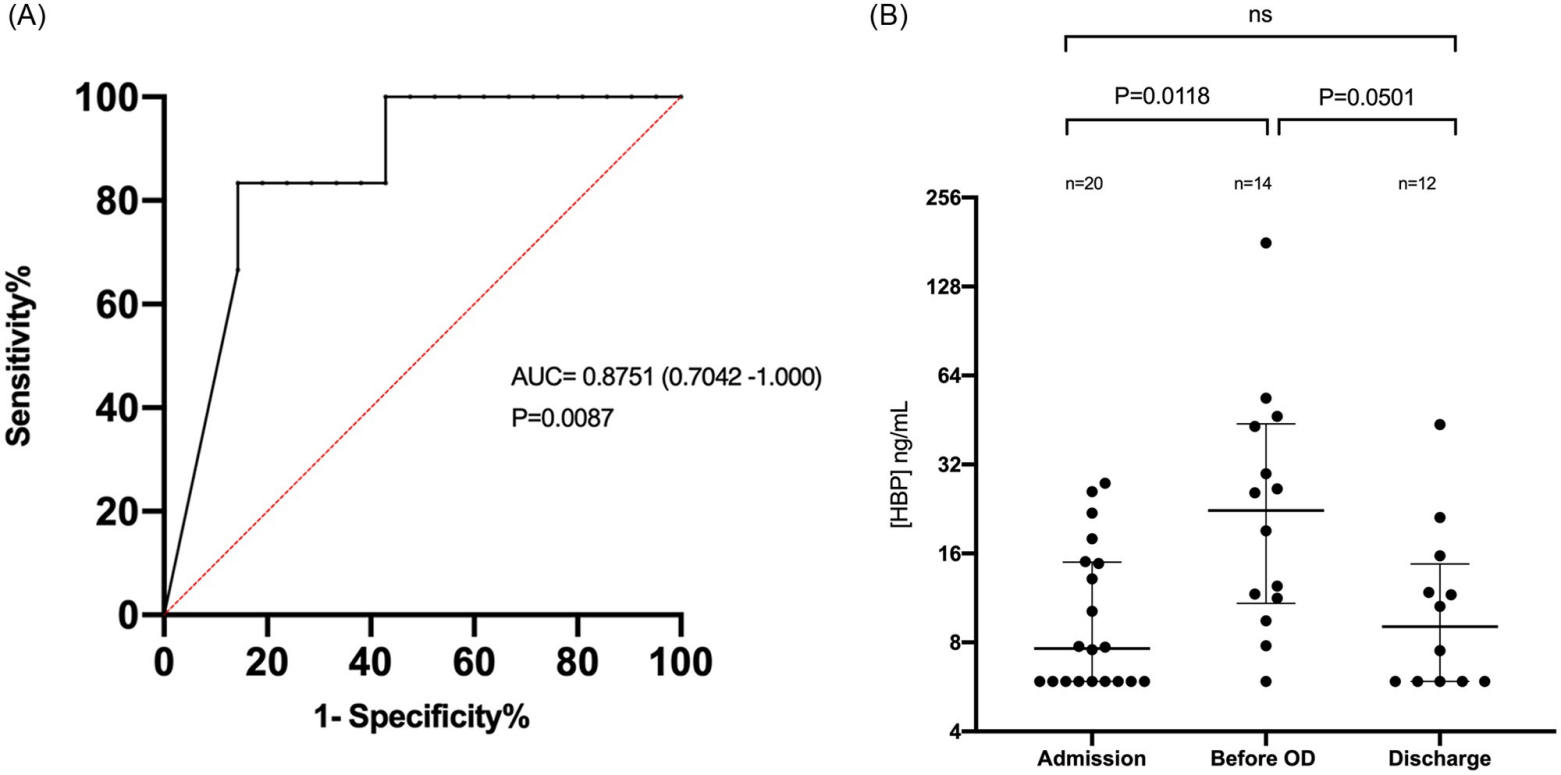
HBP prognosticates organ dysfunction in severe COVID-19. (A) A Receiver operating curve (ROC) of HBP measured by Joinstar point of care test (highest value in the first 72h) predicting organ dysfunction (OD) in severe COVID-19. Area under the curve is 0.86. (B) HBP levels in first sample taken within 72h after admission, within 48h before development of organ dysfunction, and within 48h before hospital discharge measured by the Joinstar point of care test. Samples from patients who developed organ dysfunction within 48h after admission were included in the “before OD” group instead of the “admission” group. Values were compared using Kruskal Wallis test with Dunn’s post-hoc test for multiple comparisons.

Because we found that HBP levels were elevated in patients who later developed organ dysfunction, we explored the hypothesis that HBP can predict the onset of organ dysfunction in patients with COVID-19 and that a rapid point of care assay could be useful for this measurement. We validated four technical parameters of the Joinstar point of care assay and found that it performed within specifications (Table 3) We measured plasma samples using both the HBP ELISA and the Joinstar point of care assay and found good correlation between the two with an R-value of 0.83 (Fig 2). Therefore, to explore the prognostic capacity of HBP, we used HBP values measured by the Joinstar POC test since this would be more clinically useful. A ROC curve, using the highest HBP value in the first 72 hours to predict organ dysfunction at any time, had an AUC of 0.88 (95% CI 0.70–1.0) *p*<0.01 for HBP (Fig 3A). To determine the dynamics of HBP during the course of COVID-19, we compared HBP levels in samples taken at admission, within 48 hours before onset of organ dysfunction, and before discharge from hospital. We found that HBP was significantly elevated before the onset of organ dysfunction compared to admission samples 22.4 ng/mL (IQR 10.8–43.9) vs 7.6 ng/mL (IQR 5.9–15.0), *p* = 0.01). Before discharge from hospital, median HBP was reduced to 9.0 ng/mL (IQR 5.9–14.7), p = 0.05 (Table 2 and Fig 3B).

**Table 3:** Validation of performance for Joinstar point of care test for plasma HBP.

Test item	Tested value	Target value according to the manufacturer
Accuracy	R = 100.75%	Recovery rate (R) should be within the range 85–115%
Lower detection limit	LOD <5.90ng/mL	Lower limit of detection (LOD) should be <5.90ng/mL
Linearity	r = 0.99	Correlation coefficient (r)>0.990
Precision	CV = 4.36%	Coefficient of variation (CV) ≤10%

## Discussion

Our results show that the neutrophil-derived HBP, a potent inducer of endothelial dysfunction, is elevated prior to onset of organ dysfunction in patients with severe COVID-19.

This study validates the findings of Saridaki *et al*. who also found that HBP levels are increased in severe COVID-19 and to correlate to poor outcome [18]. It also emphasizes what is seen in clinical and epidemiological studies, that COVID-19 sepsis resembles bacterial sepsis [4].

Our study adds important information on both the prognostic potential and the use of a rapid point of care test, indicating great potential for clinical use in the near future. Earlier sepsis prediction, detection and diagnostics are important to enable early treatment and adequate care of patients. In an epidemic situation, with an enormous number of infected patients, most with a mild disease, it is important for triage of patients in need of health care resources. We found that HBP predicts the onset of sepsis and organ dysfunction in COVID-19, when measured by a point of care device. Point of care testing can allow for rapid measurement of HBP within 20 minutes, and therefore is available for immediate clinical application and may provide the clinician with timely and important clinical information. Saridaki *et al*. used an interesting approach and combined two biomarkers for predicting sepsis in COVID-19. Our results confirm the potential of HBP in predicting sepsis in COVID-19, whether used alone or in combination with other biomarkers and vital signs [18].

Our results add to a growing body of evidence that neutrophil activation is an important part of COVID-19 pathophysiology and might be a reason why the clinical phenotype of severe COVID-19 has common features with bacterial sepsis [23]. Bacterial superinfections are relatively uncommon in COVID-19, with less than 10% of included patients in our cohort having positive bacterial cultures in any samples. Therefore, the elevated HBP levels in this cohort cannot be explained by the presence of bacterial infections and must be caused by the viral infection itself. We show that patients with COVID-19 have plasma HBP levels below what is seen in bacterial sepsis but higher than other viral sepsis. This might reflect differences in host responses to the SARS-CoV2 virus, bacteria, and other viruses.

HBP plays a causative role in different sepsis-induced organ dysfunctions, by inducing inflammation in kidney cells and by inducing vascular leakage which leads to lung dysfunction. It is also possible that HBP plays a causative role in the onset of organ dysfunction in COVID-19, and therefore it may be a possible therapeutic target. Heparin is a potent inhibitor of many of the detrimental effects of HBP. Many hospitals now routinely administer low-molecular weight heparin (LMWH) to hospitalized COVID-19 patients to prevent clot formation [24]. It is therefore possible that an off-target benefit of heparin administration in COVID-19 is its interference with HBP.

The main strengths of this study include the measurement of HBP using two different methods, and our verification of a point of care device for HBP that can give important and rapid information to the treating physician. Limitations of the study include the small cohort size and the heterogenous timing of plasma collection between patients. Additionally, the concomitant medications administered routinely changed during the enrollment period, with the addition of LMWH and the addition of corticosteroids. Due to the small cohort size, we were not able to use statistical methods to correct for the effects of these concomitant medications. This is merely a small exploratory study and we cannot conclude whether this is representative to the population but needs to be followed by further studies.

## Conclusions

We have shown that HBP is elevated prior to development of organ dysfunction in COVID-19 using a newly developed point of care test, and hence HBP could be used in a clinical setting as a prognostic marker for the development of organ dysfunction in COVID-19. Therefore, we suggest that a randomized controlled trial of HBP as a prognostic marker of organ dysfunction in COVID-19 is warranted.

## Supporting information

S1 File (DOCX)
