# Supplementary material for: Heparin binding protein in severe COVID-19—A prospective observational cohort study
Source: PLoS One. 2021 Apr 6;16(4):e0249570. doi: 10.1371/journal.pone.0249570 (PMC8023466; doi:10.1371/journal.pone.0249570)
Supplement: S1 File — (DOCX) [file pone.0249570.s001.docx]

**S1 File**

**Jet-iStar 800 point of care HBP assay method**

The Jet-iStar 800 point of care test includes a test device, detection buffer and calibration curve. Test devices contain HBP monoclonal antibody, goat anti-rabbit IgG and a nitrocellulose membrane found on the reaction plate. The detection buffer is supplied as pre-aliquoted tubes containing 0.15mL fluorescently labeled HBP monoclonal antibody, fluorescently labeled rabbit IgG and phosphate buffer (pH 7.4). The kit was calibrated prior to each measurement using the provided calibration curve data found as a QR code on each set of reagents.

Before analyzing the plasma samples all reagents were equilibrated to room temperature. Next, 50μL of homogenized plasma was added to the detection buffer tube and thoroughly mixed by inverting. From the detection buffer tube 100μL of mixture was added vertically to the well of the reaction plate on the test device and incubated at room temperature for 18 min. During incubation the mixture diffuses into the nitrocellulose membrane by capillary action and is captured by the paired antibody immobilized by the membrane. The sample type selected was plasma and the result was read using the “quick test” setting. The intensity of the captured fluorescent antibody was quantified by the Jet-iStar 800 analyzer and the HBP concentration automatically calculated from the calibration curve data that was scanned prior to the measurement. The range of quantification was 5.90ng/mL- 300.00 ng/mL HBP. Multiple drugs have been tested by the manufacturer for interference with HBP detection in the Jet-iStar 800, many of which show no obvious effect on the results at tested concentrations. However, some interference was found when analyzing hemoglobin (4.0mg/mL), Triglyceride (8.5mg/mL) and Bilirubin (0.2mg/mL), which could be relevant in COVID-19 patients.

Supporting table. Interfering substances Jet-iStar 800. Lower concentrations than listed in the following table have no obvious effect on the test result.

| **Drug name** | **Concentration** |
| --- | --- |
| Imipenem | 1.18mg/mL |
| Cefotaxime | 0.9mg/mL |
| Vancomycin | 3.5mg/mL |
| Penicillin G | 0.4mg/mL |
| Dopamine | 0.145mg/mL |
| Norepinephrine | 2μg/mL |
| Dobutamine | 11.2μg/mL |
| Furosemide | 0.02mg/mL |
| Heparin | 16.3IU/mL |

**Jet-iStar 800 HBP assay validation**

Where plasma samples with known concentration were required, we used stored samples from a different cohort of patients in which HBP levels were already measured.

1. Accuracy

Accuracy was assessed by measuring the HBP concentration in a plasma sample with known HBP levels the range of 280-300 ng/mL (sample A) and a second sample with known HBP levels the range of 5.9-10.0 ng/mL (sample B). Fifty microliters of sample A and 450 microlitres of sample B were mixed to obtain a diluted sample (sample C), which was also measured. Recovery rate was calculated using the formula below:

$$R=\frac{C\times(V_{0}+V\text{s})-C_{0}\times V_{0}}{Vs\times C_{S}}\times100\%$$

R—recovery rate;

VS—The volume of A;

CS—The concentration of A;

V0—The volume of B;

C0—The concentration of B;

C—The mean concentration of the mixture

A recovery rate between 85-115% was considered acceptable.

2. Lower detection limit

HBP levels were measured in a sample of purified water 20 times. The average value (M) and standard deviation (SD) were calculated. If M+2SD was below 5.9 ng/mL, the lower detection limit was considered to be acceptable.

3. linearity

Two plasma samples were selected: one sample with known HBP levels the range of 280-300 ng/mL (sample A) and a second sample with known HBP levels the range of 5.9-10.0 ng/mL (sample B). Sample A and B were mixed in the following proportions:

a) 0% sample A, 100% sample B

b) 25% sample A, 75% sample B

c) 50% sample A, 50% sample B

d) 75% sample A, 25% sample B

e) 100% sample A, 0% sample B

HBP levels in the five mixed samples were measured three times each and the average value of the three measurements was calculated (Y_i_)

The linearity correlation coefficient (r) was calculated according to the formula below:

$$\frac{n\sum x_{i}y_{i}-\sum x_{i}\sum y_{i}}{\sqrt{[n\sum x_{i}^{2}-{(\sum x_{i})}^{2}][n\sum y_{i}^{2}-{(\sum y_{i})}^{2}]}}$$

X_i_=Theoretical concentration

_Yi_=Mean concentration of determination

n= number of mixed samples

4 Precision

HBP controls were provided with the HBP kit, with three different known HBP concentrations. Each control was measured 10 times on and the average value (M) and the standard deviation (SD) were calculated. Coefficient of variation (CV) was calculated according to the formula below:

CV= SD／$\overline{X}$×100％

$\overline{X}$---Mean

SD----standard deviation

CV----Coefficient of variation%.

Coefficient of variation of below 10% for each control was considered acceptable.
